# A qualitative investigation of attitudes towards aerobic and resistance exercise amongst overweight and obese individuals

**DOI:** 10.1186/1756-0500-5-191

**Published:** 2012-04-25

**Authors:** Nicola Guess

**Affiliations:** 1 Department of Dietetics and Nutrition, Newham General Hospital, Glen Road, E13 8SL, London, UK

**Keywords:** Aerobic, Resistance exercise, Overweight, Obese

## Abstract

**Background:**

Most people are not meeting the minimal requirements for physical activity participation, particularly people who are overweight or obese. Numerous initiatives have been developed which aim to increase levels of physical activity in this group, yet little is known about their feelings towards different types of exercise. In particular, resistance exercise may offer unique benefits to people seeking to lose weight, yet no study to date has examined views of resistance exercise amongst the overweight and obese. This qualitative study examined the views and attitudes towards aerobic and resistance exercise amongst overweight and obese individuals engaged in a weight management clinic.

**Methods:**

30 overweight and obese patients comprised of 25 females and 5 males, with a mean age of 40.7 years (SD = 15.2) and mean BMI of 33.8 kg/m^2^ (SD = 7.9) were recruited from a dietetic clinic to take part in baseline focus groups and interviews to assess their views on physical activity. After selecting and participating in a 12 week aerobic- or resistance-exercise program, the participants took part in follow-up interviews. Thematic analysis was then performed on the transcribed focus group and interview data.

**Results:**

For the overweight and obese women in this study, weight loss was the primary motivation for physical activity participation. Subsequently, these women perceived a failure to lose weight as strongly affecting their motivation to continue or re-engage in physical activity. Only 3 participants selected the resistance exercise option. The view of resistance exercise as a masculine activity was a dominant theme amongst all participants. A lack of knowledge of how to perform certain exercises emerged as a barrier, but was seen by the participants as surmountable given appropriate instruction.

**Conclusions:**

The females in this study cited weight loss as a primary motivation for physical activity participation. This view must be reconciled with the existing knowledge base of physical activity requirements for successful weight loss and maintenance. Participants in this study had little awareness or experience of resistance exercise, and many were fearful of the potential risks.

## Background

Moderate physical activity on 5 or more days of the week for good health is now an established public health message [1]. Longer durations of activities of 45–90 minutes daily may be necessary to prevent weight gain, and promote long-term weight loss in those previously overweight or obese [2,3]. The majority of people in the UK do not meet even the minimal requirements [4].

Commonly-used methods to increase physical activity participation in the community include brief interventions in primary care, exercise referral schemes, pedometers and community-based exercise programmes for walking and cycling [5]. Of these, exercise referral schemes have become the most wide-spread. This is despite limited evidence of their effectiveness, including amongst the overweight and obese, who may have different needs and motivations compared to other groups [6,7].

While aerobic exercise is the most widely undertaken and well-known, resistance exercise may offer unique benefits for overweight individuals, such as increased lean muscle mass [8-10], rapid subjective improvements in appearance [11], and the requirement for rest periods between exercise sets [12]. Importantly, increased muscle strength may help prevent overuse injuries which can occur in muscles unaccustomed to physical activity [13-16]. In contrast, physical activity that includes aerobic efforts is commonly performed in longer bouts which may discourage non-exercisers and be more likely to induce overuse injuries [13].

Little is known about the attitudes of overweight and obese individuals towards these different types of activity. A cross-sectional survey on physical activity preferences amongst obese patients recruited from a dietetic clinic found that women were significantly more likely than men to try keep-fit/aerobics classes and swimming [17]. However, the study did not examine these choices in depth and recommended that “in order to improve motivation and compliance, the opinions of the patients need to be sought” [17]. Similarly, while various reviews examining adherence to exercise referral schemes have cited nominal reasons for the participants’ satisfaction or adherence – such as poor body image, personal organisation and social support– the scope of these studies did not allow contextual analysis of how such factors determine physical activity participation [18-20]. A review of these studies cited the need for an in-depth exploration of participants’ experiences [20]. It has recently been shown that overweight individuals experience more displeasure during aerobic exercise compared to their normal-weight counterparts, particularly at intensities imposed by others [14,21]. Young, healthy recreationally-trained women have demonstrated altered affective responses to imposed as opposed to self-selected resistance exercise [22]. However, no study to date has specifically examined views towards resistance exercise amongst overweight and obese subjects.

The aim of this study was to gain insight into the attitudes and beliefs of overweight and obese individuals towards both aerobic and resistance exercise.

## Method

This study took place between November 2009 and August 2010 at Newham General Hospital NHS Trust. It used a mixed-method design combining a cross-sectional descriptive study to gain insight into the socio-demographic characteristics of the sample; a 12-week exercise program to give the participants experience in order to better inform qualitative data collection, and qualitative data collection pre- and post the exercise programs.

### Recruitment

Participants attending dietetic clinics for weight management were approached about the study. To be eligible for inclusion into the study, participants had to be aged 18 years or above, and have a BMI ≥25 kg/m^2^ at the time of recruitment. In addition, the participants were recruited on a quota basis to ensure representation of pre-selected population sub-sets (Table 1). These sub-sets were selected because a review of the literature suggested they may have differing needs and views towards physical activity [23-27]. All participants completed a signed consent form prior to commencing the study. 25 of the participants were female; 5 were male. The sample was multi-faith, multi-ethnic and multi-religious, and thus represented the local diverse community. Only one of the participants (male) required a translator during his one-to-one interview. All other participants were able to communicate in English. Ethical approval was sought from the Royal Brompton & Harefield Research Ethics Committee, and approval was given by the Newham University Hospital Trust R&D Department.

**Table 1:** Participant characteristics

**Population sub-group**	**Characteristics (Mean ± SD)**
18-30 years (n = 6)	Age: 25.33 ± 3.67 years; BMI 29.33 ± 4.5 kg/m^2^; 4 females and 2 males; 3 South Asian Female
≥60 years (n = 6)	Age: 63.8 ± 2.4 years; BMI 31.5 ± 5.1 kg/m^2^; 4 females and 2 males; 0 South Asian Female
BMI ≥40 kg/m^2^ (n = 6)	Age: 37.8 ± 10.9 years; BMI 46.8 ± 5.6 kg/m^2^; 5 females and 1 male; 1 South Asian Female
South Asian Females (n = 6)	Age: 34.2 ± 8.7 years; BMI 29.5 ± 2.3 kg/m^2^
Miscellaneous (n = 6)	Age: 42.3 ± 11.4 years; BMI 31.7 ± 4.2 kg/m^2^; 6 females

### Data collection

Topic guides were developed using information and gaps in knowledge identified from previous qualitative and quantitative studies [19-27]. The topic guide was piloted to ensure the questions were understood by members of the public and yielded data relevant to the study’s aims.

### Pre-exercise program settings

While the original study design had planned to collect data via focus groups alone, practical and logistical barriers meant that the qualitative data was collected via both focus groups (n = 16) and semi-structured interviews (n = 13). The same topic guide (Table 2) was used for both the focus groups and semi-structured interviews.

**Table 2:** Topic guide pre-exercise program

**Main question**	**Probing questions**
Why is physical activity good for us?	How does it benefit our bodies?
	How does it benefit our lives?
How much physical activity is enough?	
What do you see as barriers to physical activity?	Do you do enough physical activity?
	Do you think you should do more?
	Do you have plans to do more physical activity?
	What do feel is stopping you from doing more?
	Time/Health/Family/Knowledge/Motivation
	Self-image
Tell me about your previous experience of physical activity?	Have you ever taken part in a physical activity/exercise program?
	How long did it last?
	What did you most enjoy?
	What part did you least enjoy?
	What was the easiest part?
	What was the most difficult part?
What kind of physical activity/exercise do you think you would enjoy?	
Have you ever tried resistance exercise?	Do you know what this is?
	What do you think about it?
	What benefits do you think it would provide?
	What do you find attractive about it?
	What do you find unattractive about it?

Four focus groups were held with a total of 16 participants, with 13 semi-structured interviews. The focus groups lasted one hour on average; the interviews 15 minutes. The focus groups and interviews were recorded with the participants’ permission.

### Exercise program settings

Participants were invited to select one of four 12-week exercise programs (Table 3). Both leisure-centre-based activities were classed as vigorous physical activity (>6 MET), while home-based activities were classed as moderate physical activity (3–6 MET) [28,29]. Based on physical activity guidelines from the Department of Health [1], participants were advised to aim for 20 minutes of vigorous physical activity 3 times a week or 30 minutes of moderate physical activity on 5 days per week. Participants were also able to choose a home-based or leisure centre-based program based on their lifestyle needs and personal preference. Participants could choose from one of three leisure centres based in Newham. The aim of these programs was to allow each participant to access activities they may not have tried before and to feedback on their experiences. The aerobic exercise-only or resistance exercise-only programs were decided upon in order not to impose excessive time demands on the participants. However, participants choosing the resistance exercise option were encouraged to engage in some type of aerobic exercise, usually walking. Similarly, those selecting the aerobic exercise-only option were encouraged to try a form of resistance exercise. It was made clear to each participant that they could do more if they wished. Each participant was given a full induction and personalised program by a trained Exercise Specialist. All leisure centre membership costs were covered for the participants. The resistance bands were provided by Newham Council’s Exercise Referral Team.

**Table 3:** Exercise programs

**Option**	**Type of exercises**	**Duration/frequency**
H-based AE	5 minute warm-up with stretches. Walk briskly. 5 minute cool down.	30 mins; 5 days/week
H-based RE	5 minute warm-up with stretches. Series of muscle-building exercises with resistance bands including standing bicep-curl, seated leg-press, chest and back pull-downs. 5 minute cool down.	30 mins; 5 days/week
LC-based AE	5 minute warm-up with stretches. Choice of aerobic exercise at local leisure centre, including walking/running on treadmill, use of cross-trainer, step aerobics classes, aqua-aerobics. 5 minute cool down.	20 mins; 3 days/week
LC-based RE	5 minute warm-up with stretches. Series of muscle-building exercises at leisure centre, including leg-press, free-standing squats, ham-string curl, chest-press. 5 minute cool down.	20 mins; 3 days/week

### Post exercise program interviews and focus groups

Participants were requested to attend a final focus group to evaluate their views following their 12-week program. A follow-up Topic Guide was used (Table 4). Due to participant drop-out and availability it was not possible to gather the numbers required for focus groups. The interviews were conducted in person and via telephone where necessary.

**Table 4:** Topic guide post-exercise program

**Main questions**	**Probing questions**
How did you find your experience with physical activity?	What did you like about it? What did you dislike about it? How did it affect your life? What was the easiest part about it? What was the hardest part about it?
Do you feel any different about yourself?	
Would/will you continue with this program?	If not, why not? What would make you continue, or make you think about continuing?

All the focus groups and interviews were transcribed verbatim within NVIVO8 to facilitate coding, and the creation of themes and relationships. NVIVO is a qualitative data analysis (QDA) software package.

### Data analysis

The transcripts were read through within NVIVO8 and notes taken about any statements that appeared interesting or significant. Emergent themes within each text were coded. A response or set of responses was labelled as a theme if ≥5 participants discussed it. The themes coded were examined further to identify relationships between them. These relationships were then coded. These themes and relationships were subsequently assessed along with the demographic data to identify any further relationships or patterns.

## Results

**Figure 1:**
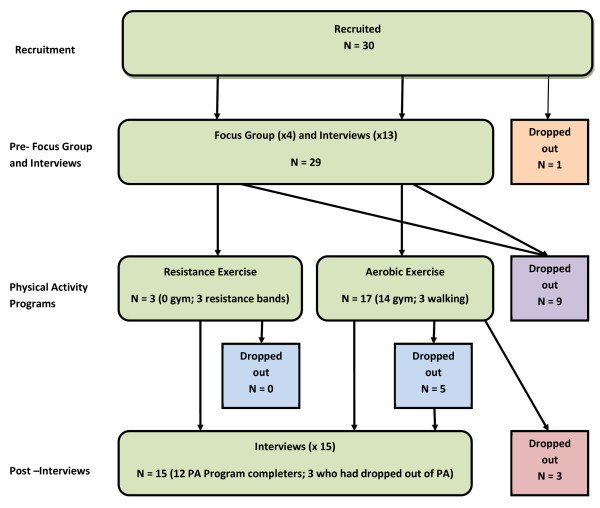
Flow-chart of participants’ progression through the study.

29 of the 30 participants enrolled took part in the initial focus groups or interviews. Of these, 20 began one of the exercise programs. 12 participants completed the 12-week programs. 15 post-interviews were held with the 12 completers (11 females, 1 male) and 3 who had dropped out during the 12 weeks. It was not possible to contact the other 14 participants. See Figure 1 for flowchart. Table 5 shows the completers and non-completers per BMI category. Analysis using Fisher’s Exact Test showed there were no significant differences in drop-out rates between BMI categories (p = .472).

**Table 5:** Exercise selection and drop-out pre- and post-exercise program by BMI category*

		**Pre-DO**^**a**^	**LC-AE**	**H-AE**	**LC-RE**	**Total**
	Overweight	4	6 (2)	1 (1)	1 (0)	12
	Obese	5	8 (5)	2 (0)	2 (0)	17
Total		9	14 (7)	3 (1)	3 (0)	29

*Figure in brackets represents number dropped-out during exercise program.

^a^ Participants who dropped out prior to selecting an exercise program.

17 people selected aerobic exercise, of whom 14 chose the leisure centre-based option. Only 3 people selected the resistance exercise option, all of whom selected home-based resistance exercise. A Chi-squared test showed the preference towards aerobic exercise to be significant (p < .000). 1 male and 2 females who had selected the leisure-centre aerobics option reported that they had tried some resistance-based exercise within the gym. The male (who had tried leisure centre-based resistance exercise prior to the study) continued with the resistance exercise in addition to his aerobic activity for the 12 weeks. The two females stopped after 2–3 visits: one female reported enjoying the aerobic classes more, while the other felt uncomfortable using the weights within a male-predominant environment.

### Views on physical activity in general

All the participants readily cited or agreed with the numerous nonweight-related health benefits of physical activity and most were able to cite the recommendations for 30 minutes moderate physical activity on 5 or more days of the week. These views were expressed in a positive and enthusiastic tone by all participants. Views encompassed included prevention of disease, reduced stress levels, and acknowledged a general ability to make us feel good. These are presented below.

"“Makes you feel good”, Female, 22 years, BMI: 25 kg/m^2^"

"“It prevents you from some diseases like high blood pressure, obesity and so many other things”, Female, 26 years, BMI: 26 kg/m^2^"

"“Yeah, I know for sure that people with good general fitness generally, tend to have a better state of mind, like less pressure”. Male, 24 years, BMI: 29 kg/m^2^"

"“Risk of diabetes. Heart disease, I think it can work towards combating a lot of those things”, Female, 24 years, BMI: 40 kg/m^2^"

"“The more physical activity you do the less ill you get, that’s basically how I see it”, Female, 34 years, BMI: 37 kg/m^2^"

In contrast, the relationship between physical activity and weight management was barely mentioned. (Two participants briefly mentioned that physical activity helps “prevent obesity”). However, when the discussions moved onto the issue of motivation, weight loss was seen as a primary motivation for nearly all the female participants. Similarly, feelings about previous experiences of physical activity were strongly determined by whether or not weight loss -or at the very least, a positive improvement in appearance - was apparent.

### Weight loss as a primary motivation

Half of the respondents stated motivation was a barrier to their exercising. As mentioned above, a strong theme amongst women was the relationship between motivation and weight, with a reduction in weight seen as the primary motivation for physical activity participation.

"“I reckon if I was a size 10 or smaller I think I would see exercise or eating healthy as a different way than I do know, I mean right …whatever I decide to do I would see it as a weight loss thing, I would see it as it is for me to lose weight, this is the only way I would see it”. Female, 24 years, BMI: 40 kg/m^2^"

"“Right, and when you’re looking at the scale, each time maybe I go to the gym, you lose 1 pounds 2 pounds, so I think afterwards, I am losing, so I think to myself I need to keep going, it’s motivating to me.” Female 64 years, BMI: 34 kg/m^2^"

"“I found that that the quickest benefits, like, you do a little bit and with your arms being toned and your legs, really quickly so it was like that’s an incentive”, Female, 26 years, BMI: 26 kg/m^2^"

"“I weigh myself every week, and see my weight. I sometimes chart my weight and seeing it physically like it helps me carry on doing what I am doing”. Female, 22 years, BMI: 25 kg/m^2^"

"“Yes, always 3 times a week, after working, losing the weight, it motivates you, makes you feel you want to do a little bit more”, Female, 61 years, BMI: 35 kg/m^2^"

"“I mean, does the person really come to the doctor and say I want to exercise because I want to be healthy or send me to the gym because I want to be healthy? No! They go on exercise and diet programs because they want to lose weight”, Female, 24 years, BMI: 46 kg/m^2^"

"“…nothing is stopping me at the moment once I start to lose a little bit of weight I start to feel motivated”, Female, 61 years, BMI: 35 kg/m^2^"

"“..but if my weight was still high, higher than the level it is supposed to be – I would not keep going”, Female, 64 years, BMI 34 kg/m^2^"

Many of these females related a lack of motivation to the feeling that weight loss was impossible:

"“If you exercise for a month you might not lose anything. You might actually gain it because you might be gaining muscle or whatever so you might not lose any weight. You might actually put something on so you think Oh my god I haven’t lost anything, I’ve gained, so then you might give up after that…and you just think you know. I can’t do this, I can’t be bothered”. Female, 24 years, BMI: 46 kg/m^2^"

"“It’s like you’re putting on weight, so you need to do something, and after a while the motivation turns to…. you stop being motivated about it, the whole thing just depresses you”, Female, 34 years, BMI: 37 kg/m^2^"

"“I find it hard to stick to [weight loss] targets because if I don’t meet them I get real down so now I try not to make such rigid targets for myself ..if it is not going to happen I am going to get de-motivated”, Female, 26 years, BMI: 26 kg/m^2^"

“So you feel like a mountain that’s kind of too big .?” “*Yeah*”, Female, 34 years, BMI: 37 kg/m^2^

This theme was strongly evident even in the post-exercise program interviews, and in many interviews it was a dominant topic in the conversation. Indeed, it was such a dominantly negative theme that in many cases it cancelled out any perceived positive benefits of physical activity. These quotes are typical:

"“I do about 20 to 30 minutes walking a day but it’s not working and it’s not making a major difference”, Female, 24 years, BMI: 40 kg/m^2^"

"“I really enjoyed it and I got into the habit of going. And towards the end my fitness level was slightly increasing…the only thing I would say is that for me was a big disappointment was that I didn’t lose a single pound. I was not expecting anything major but it was quite disappointing that I didn’t lose a single pound and I think that sort of – it kind of made me give up and I went back to my old routine. When you’re not seeing a difference you begin to question whether if it was worth it.” Female, 26 years, BMI: 26 kg/m^2^"

“What was your general experience of physical activity?”

"“It’s like banging my head against a brick wall!! I am not losing weight!” Female 65 years, BMI: 26 kg/m^2^"

None of the males in the study related physical activity to weight.

All of the 12 participants who completed the 12-week exercise program had lost weight, or explicitly stated an improvement in toning. Of the 5 participants who dropped out during the 12 weeks, 4 had not lost weight. The other had lost weight but became pregnant during the study.

### Ability and perceived ability affecting confidence

Where health or weight was discussed as a barrier, this was part of a larger theme of ability, and linked closely with the sensation of feeling different or self-conscious. For example, those who had asthma also expressed a concern at feeling different. This was no different to the concerns of those who expressed that their lack of fitness or weight might make them stand out.

"“I found it terrible in the gym. I hate it. It is isolating, and then you have all those people running for dear life and there’s sweat going all over the place, and you just walk. Very embarrassing. No, I prefer my classes”, Female 65 years, BMI: 26 kg/m^2^"

"“I feel comfortable, the problem is as soon as I feel I have problem with my asthma..” Female, 41 years, BMI: 28 kg/m^2^"

"“It’s just I suppose being with my health I have not had a lot of confidence”. Female, 42 years, BMI: 33 kg/m^2^"

"“Not really for someone like me you know with my health conditions, yeah, so I am not really sure what’s available to me”, Female, 30 years, BMI: 32 kg/m^2^"

In contrast to the discussion surrounding the issue of motivation, the discussion around ability was more positive in tone, with many participants actively questioning where they could get advice about more suitable activities for their abilities. Many of the participants were readily aware of their gaps in knowledge.

### Gaps in knowledge

12 of the participants stated that lack of knowledge was a barrier to physical activity. Responses were classed into two categories: a lack of knowledge of *how* to do physical activity and certain types of physical activity, and a lack of knowledge about *where* to do physical activity and certain types of activity. The former was consistent across all age groups and BMI categories.

"“I can’t find an exercise that isn’t painful—so that’s where I would need specialist help.” Female 21 years, BMI: 27 kg/m^2^"

"“Well due to my knee at this moment in time I do feel like that [a need for specialist advice] would maybe be the case”, Female, 30 years, BMI: 32 kg/m^2^"

"“I honestly don’t know which ones would tone up my arms say or which ones are best for me to build up my strength. You just don’t know, I mean I don’t know”, Female, 30 years, BMI: 32 kg/m^2.^"

"“Resistance exercise? Under supervision I think”, Female, 62 years, BMI: 30 kg/m^2^"

"“Well I don’t want to do myself damage, that’s what I am worried about -overdoing it”. Female, 60 years, BMI: 32 kg/m^2^"

Within these answers was an implicit suggestion that appropriate instruction could remove this barrier.

The latter was a theme which arose amongst the older participants, many of whom identified non gym-based activities as a preference:

"“Basically I asked at the local gym and my GP but neither of them knew where to go”. Female, 43 years, BMI: 34 kg/m^2^"

"“Oh if I could find a class I would do that [pilates]”, Female, 62 years, BMI: 30 kg/m^2^"

### Resistance Exercise as a Masculine Pursuit

General awareness and knowledge of this type of activity was low.

"“Most of us don’t really need weights, I don’t think”, Female, 62 years, BMI: 30 kg/m^2^"

Amongst all the participants - including those who had tried resistance exercise and those who had not - the consistent trend amongst both males and females of all ages was that resistance exercise was a masculine activity, and all females were worried about building muscles:

"“I found a lot of my family members are like ooh why are you doing that for—that’s masculine—and they try to de-feminize it. Are you trying to be a body builder and I’m just like….what?”, Female, 22 years, BMI: 25 kg/m^2^"

"“I feel that I wouldn’t want to do that, I wouldn’t want to build myself muscles. To me it’s like a man’s sport”, Female, 42 years, BMI: 33 kg/m^2^"

"“No, no, not at all, I don’t want muscles!!! I think they’re horrible!! Yeah, it’s nice to be slim and all that but not to do that no!!”, Female, 42 years, BMI: 33 kg/m^2^"

"“I don’t want big hulk muscles” Female, 26 years, BMI: 26 kg/m^2^"

Despite these concerns, all of those who had tried this kind of exercise in the past were positive about the short-term improvements in toning or strength.

"“You notice it more don’t you? I mean I could tell my arms looked different quite quick, yeah”. So you found it beneficial because you saw results quickly? “Yes” Female, 60 years, BMI: 32 kg/m^2^"

These positive statements did initially provoke the interest of other focus group members during the pre-exercise program focus groups:

"“I have a more open mind now. To the weights. Given the benefits involved. I never knew that, I mean knowledge does help, and you see things in a different light, but before I was drummed into the aerobics, so I’ll keep an open mind….” Female, 34 years, BMI: 37 kg/m^2^"

However, this did not translate in greater selection of resistance exercise when it came to selecting a 12-week program.

Even where this type of exercise was seen as positive in producing noticeable physical changes, a lack of knowledge how to perform it appropriately was seen as a barrier.

"“There are certain things you have to lift in a certain way, there are certain things, you have to be in the right position. Aerobics …it’s much easier …” Female, 34 years, BMI: 37 kg/m^2^"

"“I did use them and then now I find I have forgotten how to use them and that is the reason I don’t”, Female, 22 years, BMI: 25 kg/m^2^"

"“You don’t know which ones, I mean you’re scared you don’t want to bulk up your shoulders, I mean I honestly don’t know which ones would tone up my arms say or which ones are best for me to build up my strength”. Female,30 years, BMI: 32 kg/m^2^"

The relationship between a lack of knowledge and self-consciousness continued for this subject.

For the 3 participants who had selected the home-based resistance exercise option, all were positive about the effect it had on their appearance:

"“….it was great for my bingo arms!” Female, 38 years, BMI: 27 kg/m^2^"

"“Oh I definitely noticed an effect, really impressed”. “I also had more energy…. it made a big difference”. Female, 62 years, BMI: 30 kg/m^2^"

### Preferences or barriers within the sub-groups

#### South Asian females

Many of the younger women cited either too much encouragement/pressure to exercise (particularly from normal weight siblings), or positive support such as offers from parents to accompany them to the gym.

"“Yeah my mum goes to the gym herself so she wants me to go with her so she rags me along sometimes”, Female, 26 years, BMI: 26 kg/m^2^"

"“When I was doing [resistance] exercise at home my husband was telling me the proper moves, the proper actions”, Female, 34 years, BMI: 37 kg/m^2^"

"“My brother’s always telling me to go to the gym. My whole family is always on my case and I think that kind of makes it worse cos you think – forget it then.” Female, 22 years, BMI: 25 kg/m^2^"

"“Family is not a problem. I don’t have any children, child commitments, family commitments really”, Female, 46 years, BMI: 46 kg/m^2^"

### Participants ≥60 years old

This group were strongly responsive to the social aspect of exercise.

"“It can get a bit boring when you are on your own. I have gotten better when I am with other people doing something together—whether you are doing it as a group or whether other people are around”, Female, 62 years, BMI 30 kg/m^2^"

"“I like meeting people, and the instructors which is very, very nice. And the fact that it gets me out”, Female, 60 years, BMI: 32 kg/m^2^"

"“I could [exercise] at home. But I feel better, you know, being with other people”, Female, 62 years, BMI 30 kg/m^2^"

"“Having to meet someone there is good”, Male, 66 years, BMI 40 kg/m^2^"

"“Yes, I prefer being with other people, yes”, Female, 64 years, BMI 34 kg/m^2^"

They were also less enthusiastic generally about exercising within a gym (as mentioned above, much of this was related to perceived ability and feeling different). In contrast, the discussion became more animated when group classes such as ballroom dancing or water aerobics were mentioned. While most of the participants knew of the activities available within the Leisure Centres (which were perceived to be more aimed at younger people), only one of the participants was aware of activities available elsewhere in the community. As we discussed these other options—a collection of which are aimed solely at the ≥50 years old group - many participants expressed frustration at not being made aware of these options.

"“The only reason why I found out about the doctors referral was the physiotherapist. And then you said come here, but it has been difficult. I spent 10 years getting on the system”, Female, 65 years, BMI 26 kg/m^2^"

"“The water aerobics, there doesn’t seem to be an awful lot of it around”, Female, 65 years, BMI 26 kg/m^2^"

"“[Information] ..it’s not widely available is it?”, Female, 60 years, BMI 30 kg/m^2^"

This group also expressed concern with performing resistance exercise, and all but one (who had experience of this activity in a physiotherapy group) were worried about the risk of injury.

### Participants with BMI ≥40 kg/m^2^

Members of this group expressed feeling self-conscious while exercising. While this was not stated as an insurmountable barrier for any of the participants, it did modify the type of activity preferred.

"“I would rather work indoors, put some music on and work out myself. Cos I am too self-conscious to go to the gym with all these skinny little women”. Female, 45 years, BMI: 53 kg/m^2^"

"I feel kind of self-conscious of going to the gym or walking round the park by myself. So that’s the biggest thing for me” Male, 42 years, BMI: 43 kg/m^2^"

"“…I feel that I should have to fit in with them, I don’t care, I really don’t care what anyone else thinks about me – it’s how I feel about myself”. Female, 45 years, BMI: 53 kg/m^2^"

"“I wouldn’t feel comfortable doing it you know in a public place you know, obviously you have limitations” Female, 44 years, BMI 54 kg/m^2^"

There were no specific themes found for the 18–30 year old participants.

## Discussion

The aim of this study was to gain insight into the attitudes and beliefs of overweight and obese individuals towards both aerobic and resistance exercise. For the overweight and obese women in this study, weight loss was the primary motivation for physical activity participation. This study also found that amongst men and women of all ages, even those who have previously engaged in resistance exercise, this type of activity is viewed as a masculine one. Exploring the attitudes and motivations of overweight and obese individuals towards physical activity is critical in understanding the low participation rates amongst this group.

It is now clear that durations of physical activity in the order of 60–90 minutes per day are required for successful weight loss and maintenance [2,3]. When discussing their previous experiences of physical activity, many of the women in this study reported discontinuing their exercise programs (generally of about 30 minutes duration) when weight loss was not forthcoming. Additionally, as mentioned above, of the 5 participants who dropped out of the exercise program during the 12 weeks, 4 had not lost weight.

It has been acknowledged that people may be discouraged by advice to exercise for 60–90 minutes per day [30]. However, by advising overweight individuals to aim for 30 minutes physical activity, it is possible the subsequent lack of weight loss may actually prove a negative reinforcement for physical activity participation, at least in females. This would suggest that health practitioners and public health bodies must tactfully manage patient and public expectations of physical activity.

While none of the males in this study openly shared the same view, a recent European Union-wide survey found that a quarter of respondents (both male and female) thought that “unless physical activity resulted in weight loss, they were not really benefiting from it” [31].

It was unfortunate that only five males were recruited for this study. Since men dissatisfied with their body shape are more likely to exercise than women [23], it would be interesting to explore whether this is because men believe more strongly than women that physical activity will have a positive effect on their body shape. It has been suggested that women see the ideal body shape as “slim”, whereas men would like to be “muscular” or “fit” [6,32]. As the effect of physical activity on muscle tone and fitness occurs relatively quickly compared to the effect of physical activity on weight loss, it is feasible that overweight men are more likely to believe physical activity will help them achieve their desired body shape, whereas women do not. This is a question that should be explored further.

Consistent with the findings amongst the general population, knowledge and awareness of resistance exercise in this population was very low [33]. Moreover, the belief that this resistance exercise was for men only permeated each discussion. While advice to increase physical activity in general has been available for decades, muscle-strengthening exercises were only explicitly included in the American College of Sports Medicine guidelines in 2007 [34]. It is therefore not surprising that the awareness of -and knowledge of the benefits of – this type of activity is low. Those participants who had tried this type of activity prior to the exercise programs did acknowledge rapid improvements in muscle toning, which they saw as positive; however, this did not translate into greater selection of this activity by the participants. 14 out of 17 (82%) females and 3/3 (100%) males chose the aerobic option, demonstrating a significant preference for aerobic exercise. While it’s clear from the pre-exercise program focus groups and interviews that the participants were concerned about the risk of injury, how to perform the exercise correctly and unwanted muscle development, feedback from a greater number of participants choosing the resistance option could have provided more insight.

A study examining predictors of resistance versus aerobic exercise among male and female university students found that 40% of those participating in resistance exercise did so to lose weight, compared to 57% of those participating in aerobic exercise [27]. Moreover, 22% of those participating in resistance exercise stated they did so to gain weight. The expected effect of resistance exercise on body weight among the general public is thus not clear and may explain why individuals anxious about their weight may avoid it. The researchers also found that nearly half of those who did not participate in resistance exercise said it was because they either did not know how (46%) or because they did not want to become “too bulky” (48%) [27]. This study replicated these findings. It seems clear that more public health education on the realistic benefits, both physical and physiological, would be helpful to dispel some of the myths surrounding resistance exercise.

None of the participants were aware of a proposed beneficial effect on body composition. The evidence supporting improvements in lean muscle mass (or amelioration of loss of lean body mass during diet-induced weight loss) is somewhat contradictory; however, this may be due to a heavy load being required to significantly alter body composition, which can usually only be achieved in a gym setting [35]. Nonetheless, the fact that resistance exercise is the second most common exercise undertaken by “successful [weight] losers” in the National Weight Control Registry indicates the potential contribution this type of activity could make to healthy weight management [36]. A dedicated qualitative examination of attitudes to evidence-based prescriptions of resistance exercise would be valuable to this end.

The evidence for recommending muscle-strengthening exercises for older individuals is very strong [33,37,38]. It is therefore significant that this sub-population were immediately concerned about the risk of injury of this type of activity. These concerns represent a clear opportunity for qualified exercise professionals to assist and guide this group in performing this potentially highly-beneficial exercise safely.

Consistent with other papers examining attitudes to physical activity amongst the obese, a majority of the participants with a BMI ≥40 kg/m^2^ in this study reported feeling self-conscious about their weight when exercising, and modifying their activities accordingly. Perceived disapproval of body shape – known as social physique anxiety – has been found to be a barrier to physical activity participation in both young and old obese adults, and a particularly difficult experience for obese adults is to exercise in the company of “super-fit” gym members [39,40]. This view did not emerge as strong a theme as expected amongst the obese participants in this study, which may be partly due to the unintended focus group composition (see below). Perhaps tellingly, the 2 obese participants who completed the 12-week program had chosen a walking program where they could exercise where they felt comfortable, and at their own pace. 2 females (both aged 18–30 years, BMI ≥ 40 kg/m^2^) selected the leisure centre-based aerobic option and dropped out 3 weeks into the 12-week program. They both took part in post-exercise program interviews where they offered a variety of reasons for not continuing, including time, knee pain, “forgetting to go” and difficulties with transport. Both, however, stated that they felt comfortable in the gym-environment.

The participants aged ≥60 years had very distinct preferences to the 18–30 years group. This included a desire for activities to be social and a preference for non gym-based activities. Some of the younger participants were dismissive of classes they considered for “older people”. Equally some of the older participants were worried about doing more high-intensity classes and not keeping up. While the topic guide did not ask specifically whether people preferred exercising within their own age-group, this has been suggested as a recommendation previously [41]. Such practical arrangements could easily be made at low-cost and at a local level, and could reduce barriers to physical activity participation.

Similarly, the gaps in knowledge identified by the participants, such as accessing different types of exercise classes, could be met by targeted promotion by the facilities which run these classes.

Previous studies with a qualitative component have identified specific barriers to physical activity participation amongst females of South Asian origin, particularly Muslim women [26,42]. These include a need for an all-female exercise environment and a lack of support from family members. For this reason, this study sought to examine the views of this sub-population within an all-female focus group, and a question on family as a barrier to physical activity was added to the topic guide. These issues did not emerge as a theme amongst any of the South Asian females in this study. One of the females stated that she preferred an all-female environment, but this was not a necessity. The difference may be explained by the high educational level and high-English proficiency of the females in this study, which has been noted previously [29].

The aim of this study was to explore the attitudes of overweight and obese individuals to aerobic and resistance exercise. The study design required the participants to select either aerobic or resistance exercise programs in order to avoid imposing excessive time demands on the participants. However, the participants were encouraged to try other types of exercise and were provided with a 3-month Leisure Centre pass and guidance with using the Dynabands in order to support this aim. It was not expected that the subjects would significantly favour the aerobic exercise option, particularly in an environment where they were given individual instruction. As such, the question as to why the participants did not choose to try resistance exercise was not added to the topic guide. While the fact that so few subjects tried resistance exercise limited the qualitative data that could be gathered in this study, it is also a significant finding in itself.

While this study sought to recruit to quota to ensure the sample was representative of the local community, this had unintended consequences. No quota was set for the recruitment of males as it was expected based on a review of the literature that men would more readily take part than females. Unfortunately, it was not possible to recruit more males for this study in the time given. While the men in this study did contribute fruitfully to the discussions, it is understandable that on many occasions this was to respond to a female-led point of view. Notably, however, none of the males in this study mentioned weight loss as a primary motivation for physical activity participation, despite it being a dominant theme in the discussions. Although only 2 of the 5 males in this study had previously tried resistance exercise, these individuals were both ≤30 years. It is likely that a large group of younger males would have a more positive view of this type of exercise.

All participants were requested to take part in a final interview even if they dropped out of the study at any point. This was to ensure that all views were captured and to prevent attrition bias. 9 subjects dropped out prior to, and 8 people dropping out during the exercise programs. Unfortunately, only 3 subjects who had dropped out during the exercise programs completed a post-exercise program interview. Although efforts were made to contact the 9 participants who dropped out prior to starting, it was not possible. Although the views of these individuals were collected in the initial focus groups and interviews, their reasons for not completing the study would be as informative as the data collected from the completers.

While it was intended that each focus group would be comprised solely of individuals from the same population sub-set, practical limitations (weather, job commitments) meant that two of the focus groups were of mixed composition. It is therefore possible that particular participants may have felt uncomfortable during the recording, and felt there were certain views they could not express. Each of the discussions was animated and very enthusiastic in parts, and while it was not readily apparent that any of the participants appeared uncomfortable or reticent, this of course cannot be discounted as a possibility.

While a sample of 30 participants is considered large for qualitative research, the variety of different views which became apparent in the recording of the focus groups and interviews indicated that similar work could be conducted on a more narrowly-defined sample in order to refine the data produced by this study.

## Conclusions

The females in this study cited weight loss as a primary motivation for physical activity participation. This view – if this finding is replicated in further studies—must be reconciled with the research conducted by expert bodies that clearly demonstrates that larger durations of physical activity are required if successful weight management is desired.

Participants in this study had little awareness or experience of resistance exercise, and many were fearful of the potential risks. Greater public education on the benefits of resistance exercise—particularly for older individuals - would help dispel some of the myths surrounding this activity.

Both national and international surveys demonstrate that individuals who are overweight and obese are less likely to participate in physical activity. Encouraging participation must take into account the differing needs of this heterogeneous population. Local exercise referral pathways or interventions must therefore ensure a range of attainable and sustainable options are both available and well-advertised.

## Competing interests

The author declares that they have no competing interests.
